# Rapid detection of *Streptococcus agalactiae* using an RPA-MbCas12a CRISPR assay targeting novel *sodA* and *groL* markers

**DOI:** 10.3389/fmicb.2026.1918484

**Published:** 2026-08-24

**Authors:** Aldan Shamukhan, Aisha Shaizadinova, Meruyert Amanzholova, Pavel Tarlykov, Sailau Abeldenov

**Affiliations:** 1 National Center for Biotechnology, Astana, Kazakhstan; 2 Faculty of Biology and Biotechnology, Al-Farabi Kazakh National University, Almaty, Kazakhstan

**Keywords:** Cas12a, CRISPR diagnostics, point-of-care detection, RPA, *Streptococcus agalactiae*

## Abstract

*Streptococcus agalactiae* (Group B *Streptococcus*, GBS) is a major cause of neonatal and invasive infections, necessitating rapid and sensitive diagnostic approaches beyond conventional culture and PCR. Here, we developed an isothermal recombinase polymerase amplification (RPA)-MbCas12a CRISPR assay for fluorescence-based detection of GBS using a recently characterized MbCas12a variant from *Moraxella bovoculi*. The assay targets two conserved housekeeping genes, *sodA* and *groL*, and operates under isothermal conditions at 37 °C with minimal sample processing. Analytical sensitivity reached 1.6 × 10^1^ to 2.5 × 10^1^copies per reaction, while analytical specificity was confirmed against non-target bacterial species. The assay successfully detected all 14 tested GBS isolates directly from crude heat lysates and generated results within 1 h. By combining rapid isothermal amplification, simplified sample preparation, and MbCas12a-mediated detection, this platform represents a promising approach for point-of-care GBS screening and infection control in resource-limited settings.

## 1 Introduction

Group B *Streptococcus* (GBS), also known as *Streptococcus agalactiae*, is a globally important pathogen affecting both humans and animals. In humans, GBS asymptomatically colonizes the rectovaginal and gastrointestinal mucosae of up to 30% of healthy adults, typically acting as a commensal organism. However, it remains a major cause of invasive disease in vulnerable populations, including neonates, pregnant women, older adults, and immunocompromised individuals (Raabe and Shane, 2019). Historically, GBS was first described in veterinary contexts, particularly as a cause of bovine mastitis responsible for substantial economic losses in dairy herds; its species name, *agalactiae*, refers to the cessation of milk production observed in infected cattle (Keefe, 1997). The dual nature of GBS as both a harmless commensal and a potent pathogen reflects its versatile pathogenic repertoire, which includes a polysaccharide capsule, adhesins, invasins, and other virulence factors that facilitate tissue invasion and immune evasion (Armistead et al., 2019; Gergova et al., 2024).

Group B *Streptococcus*is most prominently recognized as a leading cause of neonatal sepsis, pneumonia, and meningitis. However, its clinical significance also extends to non-pregnant adults, particularly in hospital and healthcare settings, where it contributes to bloodstream infections, pneumonia, surgical site infections, and other severe complications (Crespo-Ortiz et al., 2014; Seale et al., 2017; Madrid et al., 2017). Nosocomial GBS infections may arise through transmission from colonized patients, healthcare workers, or contaminated clinical environments, although the exact pathways of in-hospital transmission remain incompletely characterized (Collin et al., 2019; Plainvert et al., 2020). Outbreak investigations and clinical surveillance have documented clusters of invasive Group B Streptococcal disease within acute care facilities, underscoring the need for robust diagnostic and infection control strategies (Collin et al., 2019; Barkham et al., 2019).

The ecological breadth of GBS extends well beyond human hosts. Molecular epidemiological studies have revealed the presence of genetically related subpopulations of GBS across a diversity of animal species, including aquatic mammals and fish (Delannoy et al., 2013). Certain sequence types (e.g., ST283) associated with invasive disease in humans have been recovered from fish, and epidemiological linkages suggest that contact with or consumption of contaminated aquatic products can facilitate cross-species transmission events (Li et al., 2025). Comparative genomic analyses further demonstrate close evolutionary relationships between human and piscine isolates, indicating that some strains have broad host tropism and the potential for zoonotic or reverse-zoonotic transmission (Liu et al., 2013; Kawasaki et al., 2018). Such findings reinforce the conceptual framework of One Health, wherein human, animal, and environmental reservoirs interact in the circulation and evolution of pathogens like GBS (Meroni et al., 2022).

In addition to piscine reservoirs, GBS has been isolated from various terrestrial mammals, including livestock and camelids, further illustrating the bacterium’s wide host range (Tavella et al., 2018; Carra et al., 2021; Zhao et al., 2022). These observations raise important questions about the role of animal reservoirs and environmental sources in the epidemiology of GBS infections in humans and the potential for transmission into clinical settings. The zoonotic potential of GBS is supported by genomic and phenotypic evidence showing shared virulence determinants and antimicrobial resistance profiles between animal and human isolates (Hassan et al., 2025).

From a clinical microbiology and diagnostic perspective, efficient and accurate detection of GBS remains essential for guiding patient management, infection prevention, and public health surveillance. Traditional culture-based methods using blood agar and selective media such as Granada medium enable recovery of *β*-hemolytic GBS colonies, sometimes supplemented by confirmatory tests like the CAMP reaction (Rosa-Fraile and Spellerberg, 2017; Filkins et al., 2020). However, culture can be time-consuming and may yield false negative results (Gao et al., 2021). The incorporation of molecular techniques such as qPCR targeting conserved GBS genes has improved sensitivity and turnaround time, facilitating early diagnosis and outbreak detection in contexts (El Helali et al., 2009; Mazlumoğlu et al., 2026). Ongoing research efforts are focused on the development and evaluation of advanced molecular assays, including rapid and point-of-care platforms, reflecting continued interest in improving diagnostic approaches across diverse clinical populations.

In recent years, CRISPR-based diagnostics have emerged as a highly effective approach for nucleic acid detection, attracting increasing attention due to their specificity, programmability, and suitability for rapid and point-of-care applications. These systems rely on CRISPR-associated nucleases that, guided by a complementary RNA, bind to target nucleic acid sequences and introduce site-specific cleavage. In the case of Cas12a, recognition and cleavage of double-stranded DNA induce a conformational activation of the enzyme, which subsequently acquires non-specific single-stranded DNase activity. This collateral (trans) cleavage enables the degradation of reporter oligonucleotides, resulting in a measurable signal and providing a basis for sensitive detection (Chen et al., 2018).

To further enhance sensitivity and enable detection of low-abundance targets, CRISPR-based assays are frequently combined with isothermal nucleic acid amplification techniques. Recombinase polymerase amplification (RPA) is especially well suited for this purpose, as it operates at constant low temperatures (37–42 °C), significantly reducing assay time and eliminating the need for thermocycling equipment (Lobato and O'Sullivan, 2018). The integration of RPA with CRISPR detection thus provides a synergistic approach that combines rapid amplification with highly specific target recognition, while maintaining compatibility with decentralized diagnostic settings (Gootenberg et al., 2017; Kaminski et al., 2021). Accordingly, several CRISPR-based diagnostic assays for GBS have been developed, primarily focusing on prenatal screening applications in pregnant women, where rapid identification of GBS colonization is critical for preventing neonatal disease (Yu et al., 2023; Zheng et al., 2024; Li et al., 2025). These approaches demonstrate the strong diagnostic potential of CRISPR-based platforms for nucleic acid detection in clinically relevant settings. Considering the genetic diversity of GBS, broader evaluation of assay performance across different clinical settings remains important. In this context, the present study contributes to the ongoing development of CRISPR-based diagnostics by expanding the repertoire of molecular targets available for GBS detection through the evaluation of two conserved housekeeping genes in clinical isolates.

The applicability of RPA–CRISPR diagnostics has recently expanded to a broad range of clinically important bacterial pathogens, particularly those associated with healthcare settings. Recent studies have demonstrated rapid and highly sensitive CRISPR-based detection of major hospital-associated pathogens, including *Klebsiella pneumoniae*, *Acinetobacter baumannii*, *Staphylococcus aureus*, and *Mycoplasma pneumoniae*, highlighting the versatility of combining isothermal amplification with Cas12-mediated detection across diverse bacterial species (Wu et al., 2025; Liao et al., 2026; Li et al., 2025). These studies demonstrate the versatility of CRISPR-based diagnostics across diverse bacterial pathogens and provide a broader context for the development of new CRISPR-based detection strategies, including the evaluation of alternative molecular targets and CRISPR nucleases.

In the present study, we expand the molecular diagnostic toolbox for GBS by developing an RPA–CRISPR assay based on MbCas12a (*Moraxella bovoculi*), a Cas12a ortholog distinguished by robust trans-cleavage activity and applicability in nucleic acid detection. To enhance assay reliability, we targeted two conserved housekeeping genes, *groL* and *sodA*, which have been widely applied in phylogenetic and taxonomic analyses of streptococci because they contain sufficient species-specific sequence polymorphisms for reliable discrimination of closely related *Streptococcus* species. These characteristics have also supported their application in molecular identification assays, yet they have not previously been employed in CRISPR-based detection of GBS (Poyart et al., 1998; Glazunova et al., 2009; Leigh et al., 2018; Cheng et al., 2020). By combining MbCas12a with a dual-gene detection strategy, this work expands the range of molecular targets available for CRISPR-based GBS detection and provides an analytical framework for the development of future CRISPR-based diagnostic assays while maintaining compatibility with rapid isothermal amplification and simplified sample preparation.

## 2 Materials and methods

### 2.1 Oligonucleotides

The following oligonucleotides were used in the work (Table 1).

**Table 1:** Oligonucleotides.

Method	Oligonucleotide	Sequence (5’ → 3’)
RPA	sodA-Fw	TAACCACGCTCTTTTCTGGGAATTGATGTC
	sodA-Rv	GCTTTGATGTAGTTAGGACGAACATTACGA
	groL-Fw	CGTGGGTAATGATGGTGTTATCACTATTGA
	groL-Rv	CGAATTTTGTTAAGAACAAGCGTTGGGAGA
*In vitro* transcription	MbCas12a-crRNA-sodA-compl	CAGCTTTAAAGTCTTCAAATGAACATCTACAAACAGTAGAAATTCCCTATAGTGAGTCGTATTAGAATT
	MbCas12a-crRNA-groL-compl	TATACTGTGACAAGTACCCACGGTATCTACAAACAGTAGAAATTCCCTATAGTGAGTCGTATTAGAATT
	MbCas12a-crRNA-SHORT	AATTCTAATACGACTCACTATAGGG
Fluorescence	ssDNA Reporter	FAM-TTATT-BHQ-1

### 2.2 Design of crRNAs

In this study, crRNAs targeting the *sodA* and *groL* genes were designed based on sequences retrieved from the NCBI database (Gene ID: 66885740 and Gene ID: 66886814). Genomic regions of 312 bp (*sodA*) and 300 bp (*groL*) were used as templates for spacer selection. For both targets, 24-nt spacers were selected immediately downstream of the Cas12a-compatible PAM sequence (TTTG) to ensure specific and efficient target recognition.

### 2.3 crRNA synthesis

crRNAs were synthesized using the HiScribe™ T7 Quick High Yield RNA Synthesis Kit (New England Biolabs). Synthetic DNA oligonucleotides of varying lengths were used as templates for *in vitro* transcription. Each template contained a double-stranded T7 promoter region upstream of the transcription sequence. The T7 promoter sequence used was 5′-TAATACGACTCACTATAGGG.

Templates were generated by annealing a short oligonucleotide (10 μM) with its complementary oligonucleotide (10 μM) by incubation at 75 °C for 3 min, followed by gradual cooling to room temperature over 1 h. The resulting double-stranded oligonucleotide duplex served as the transcription template.

Following transcription, RNA was purified using the Monarch® RNA Cleanup Kit (New England Biolabs) with an additional DNase I treatment to remove residual DNA templates, according to the manufacturer’s instructions. RNA concentration was measured using a NanoDrop spectrophotometer (Thermo Scientific), and RNA samples were diluted to working concentrations with nuclease-free DEPC-treated water. Purified crRNAs were used immediately or stored at −80 °C until use.

### 2.4 Preparation of positive control templates

For the generation of positive controls, the *sodA* and *groL* genes were amplified by PCR using Phusion DNA polymerase with primer pairs identical to those used for the RPA reaction. The resulting PCR products of 312 bp (*sodA*) and 300 bp (*groL*) were verified and subsequently cloned into plasmid vectors using the CloneJET PCR Cloning Kit (Thermo Scientific). The obtained plasmids were sequenced to confirm insert integrity and the absence of mutations. These plasmids were then used as positive control templates for optimization of the isothermal RPA reaction and for analytical sensitivity assessment of the assay.

### 2.5 Expression and purification of recombinant MbCas12a

Recombinant MbCas12a was expressed from a plasmid encoding the MbCas12a gene, previously constructed and validated in our laboratory. Protein expression and purification were performed according to a protocol described earlier by our group (Shaizadinova et al., 2023; Akimbekova et al., 2026), with minor modifications. Briefly, Cas12a was expressed in *E. coli* ArcticExpress (DE3), followed by cell lysis and purification using affinity chromatography. The purity and integrity of the recombinant protein were assessed by SDS-PAGE. Purified MbCas12a was aliquoted and stored at −80 °C until further use in CRISPR/Cas12a assays.

### 2.6 RPA reaction conditions

Recombinase polymerase amplification was performed using the TwistAmp Basic Kit (TwistDx, UK). The reaction mixture was prepared by combiningthe corresponding forward and reverse primers listed in Table 1 (sodA-Fw/sodA-Rv or groL-Fw/groL-Rv), primer-free rehydration buffer, and nuclease-free water, followed by reconstitution of the lyophilized RPA enzyme pellet with magnesium acetate to initiate the reaction. The resulting master mix was aliquoted into 9 μL portions, after which 1 μL of template DNA was added to each reaction. Although the manufacturer recommends an operating temperature range of 37–42 °C for TwistAmp reactions, amplification was carried out under isothermal conditions at 37 °C for 30 min to allow the subsequent MbCas12a detection step to be performed under the same incubation conditions, thereby enabling the entire workflow to be carried out under a single isothermal condition without changing the incubation temperature.

### 2.7 RPA-CRISPR/Cas12afluorescence assay

To assemble MbCas12a–crRNA ribonucleoprotein (RNP) complexes, MbCas12a and the corresponding crRNA were incubated at equimolar concentrations at room temperature for 15 min. The MbCas12a trans-cleavage reaction was performed in a final volume of 30 μL containing NEB buffer 2.1 (New England Biolabs), MbCas12a and crRNA at final concentrations of 100 nM each, a fluorescent single-stranded DNA reporter (5 μM), and 3 μL of RPA amplification products(10% of the final reaction volume), a proportion selected to provide sufficient amplified template while minimizing the carryover of RPA reaction components into the CRISPR detection step. The reaction mixture was incubated at 37 °C for up to 30 min. Fluorescence signals were recorded under UV illumination at 320 nm using a Vilber Lourmat transilluminator (France), and images were captured at 10, 20, and 30 min using a smartphone camera for qualitative analysis.

### 2.8 Real-time fluorescence readout

Real-time fluorescence measurements were performed using a CFX96 Real-Time PCR System (C1000 Touch Thermal Cycler, Bio-Rad). Fluorescence intensity was recorded at 1-min intervals over a 30-min reaction period.

### 2.9 Evaluation of analytical sensitivity and specificity of the RPA–CRISPR/Cas12a assay

The analytical sensitivity of the assay was evaluated using ten-fold serial dilutions of plasmid DNA containing the *sodA* or *groL* target fragments. Plasmid copy numbers were calculated using the standard equation:

$$\text{Copies}/\mathrm{\mu L}=(\begin{array}{l} \mathrm{DNA}\;\text{concentration}\;(g/\mathrm{\mu L})\\ \times 6.022\times 10^{23} \end{array})/(\begin{array}{l} \text{plasmid length}\;(\mathrm{bp})\\ \times 660 \end{array})$$

Where 6.022 × 10^23^ is Avogadro’s constant and 660 g/mol represents the average molecular weight of one base pair of double-stranded DNA. The total plasmid sizes were 3,286 bp for the *sodA* construct and 3,274 bp for the *groL* construct. Initial plasmid DNA concentrations, determined using a NanoDrop spectrophotometer (Thermo Scientific), were 58.3 ng/μL for the *sodA* plasmid and 91.2 ng/μL for the *groL* plasmid. Based on these values, plasmid stocks were diluted to initial concentrations of 1.6 × 10^10^ and 2.5 × 10^10^ copies per reaction for the *sodA* and *groL* assays, respectively, followed by ten-fold serial dilutions down to 10^0^ copies per reaction. The analytical LoD was operationally defined as the lowest target concentration producing a fluorescence signal above the predefined positivity threshold. Assay specificity was assessed using genomic DNA from non-target bacterial species commonly associated with healthcare-associated infections, including *Escherichia coli*, *Staphylococcus aureus*, *Klebsiella pneumoniae*, and *Acinetobacter baumannii*.

### 2.10 Evaluation of bacterial samples

The assay was evaluated using 14 farm-derived GBS isolates obtained through an academic collaboration. The isolates had been previously identified as *S. agalactiae* by the providing laboratory before their inclusion in this study. They were selected for analytical evaluation because of the broader One Health relevance of animal-associated GBS populations.

For rapid and low-resource template preparation without reliance on commercial DNA extraction kits, bacterial colonies were collected directly from agar plates using a sterile loop, resuspended in 100 μL of nuclease-free water, and lysed by heating at 95 °C for 5 min. After centrifugation at 10,000 × g for 2 min, the crude supernatant was used undiluted as the template for RPA amplification targeting the *sodA* and *groL* genes, followed by CRISPR–Cas12a detection.

## 3 Results

### 3.1 Schematic overview of the RPA-CRISPR/Cas12a detection strategy

**Figure 1:**
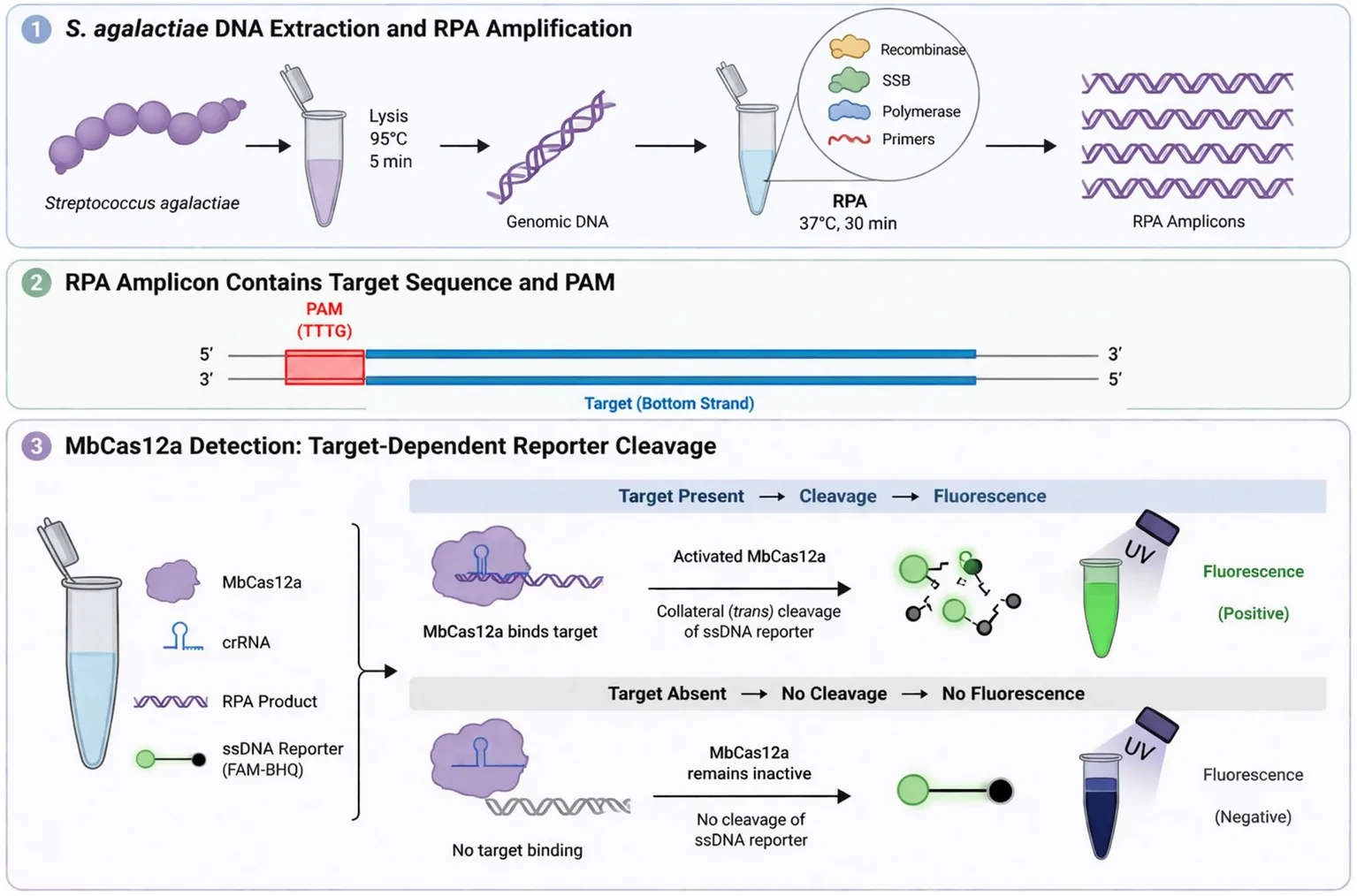
Schematic representation of the RPA–CRISPR/Cas12a assay for detection of GBS. Bacterial cells undergo heat lysis (95 °C, 5 min) to release genomic DNA, followed by RPA amplification at 37 °C for 30 min. The resulting amplicons contain the target sequence and a TTTG PAM site required for Cas12a recognition. Detection is performed by incubating the RPA products with MbCas12a, crRNA, and a fluorescent ssDNA reporter. In the presence of the target, Cas12a is activated and cleaves the reporter, producing a fluorescent signal under UV illumination. In the absence of the target, no reporter cleavage occurs and no fluorescence is detected.

This study employs a two-stage RPA–CRISPR/Cas12a workflow for rapid and specific detection of GBS. Initially, bacterial cells are subjected to rapid heat lysis to release genomic DNA, which is subsequently amplified using recombinase polymerase amplification (RPA) under isothermal conditions. The resulting amplicons are designed to include both the target sequence and a protospacer adjacent motif (PAM), enabling recognition by the MbCas12a–crRNA complex. Upon target binding, MbCas12a is activated and induces nonspecific trans-cleavage of a fluorescent ssDNA reporter, generating a detectable signal. In the absence of the target sequence, the enzyme remains inactive and no fluorescence is observed, allowing clear discrimination between positive and negative samples (Figure 1).

### 3.2 Analytical specificity of the RPA–CRISPR assay

**Figure 2:**
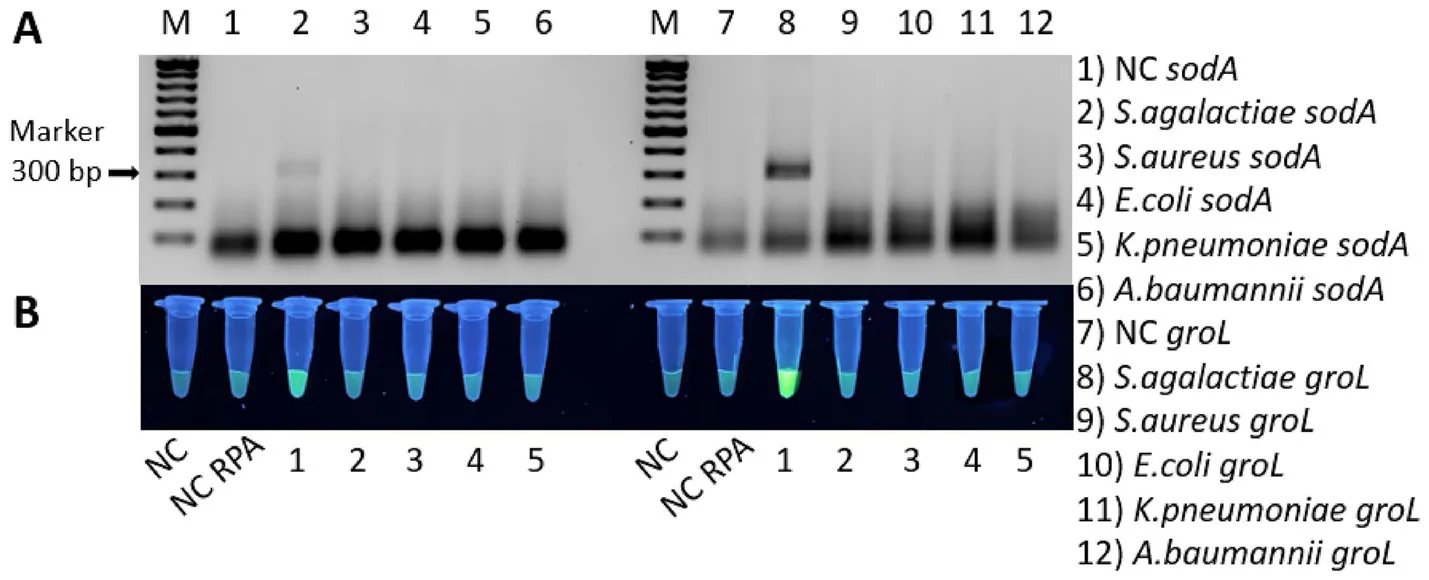
Analytical specificity of the RPA-CRISPR assay for GBS detection. **(A)** Agarose gel electrophoresis of RPA products obtained using *sodA* and *groL* gene targets from genomic DNA of the indicated bacterial species. **(B)** Fluorescence readout of the corresponding CRISPR-MbCas12a detection reactions.

Multiple sequence alignments of the selected target regions and RPA primer binding sites were performed during assay design to identify variable regions allowing discrimination of GBS from related bacteria. Additionally, alignments of the *groL* and *sodA* target regions across strains representing the most prevalent GBS serotypes (Ia, Ib, II, III, and V) revealed a high degree of sequence conservation (Supplementary Figure S1). The multiple sequence alignments included both the RPA primer-binding regions and the MbCas12a crRNA target sites. The crRNA target regions contained multiple mismatches with non-target bacteria, with ≥5 mismatches observed for the *groL* target and ≥4 mismatches for the *sodA* target (Supplementary Figures S2, S3). Together with the analyzed primer-binding regions, these alignments support the analytical specificity observed experimentally. The analytical specificity of the RPA-CRISPR assay was assessed using genomic DNA from GBS and non-target bacterial species, including *Staphylococcus aureus*, *Escherichia coli, Klebsiella pneumonia* and *Acinetobacter baumannii* (Figure 2). Two conserved target genes, *sodA* and *groL*, were evaluated to determine whether the assay selectively amplifies and detects GBS without cross-reactivity toward phylogenetically or clinically relevant bacteria.

As shown in Figure 2, RPA amplification yielded detectable products only for GBS templates for both *sodA* and *groL* targets, while no amplification was observed for *S. aureus* or *E. coli* (Figure 2A). Subsequent CRISPR-mediated fluorescence detection confirmed these results, with a fluorescent signal detected exclusively in reactions containing GBS amplicons (Figure 2B). No fluorescence above background was observed for non-target species, indicating high analytical specificity of the assay for GBS detection.

### 3.3 Analytical sensitivity and limit of detection

**Figure 3:**
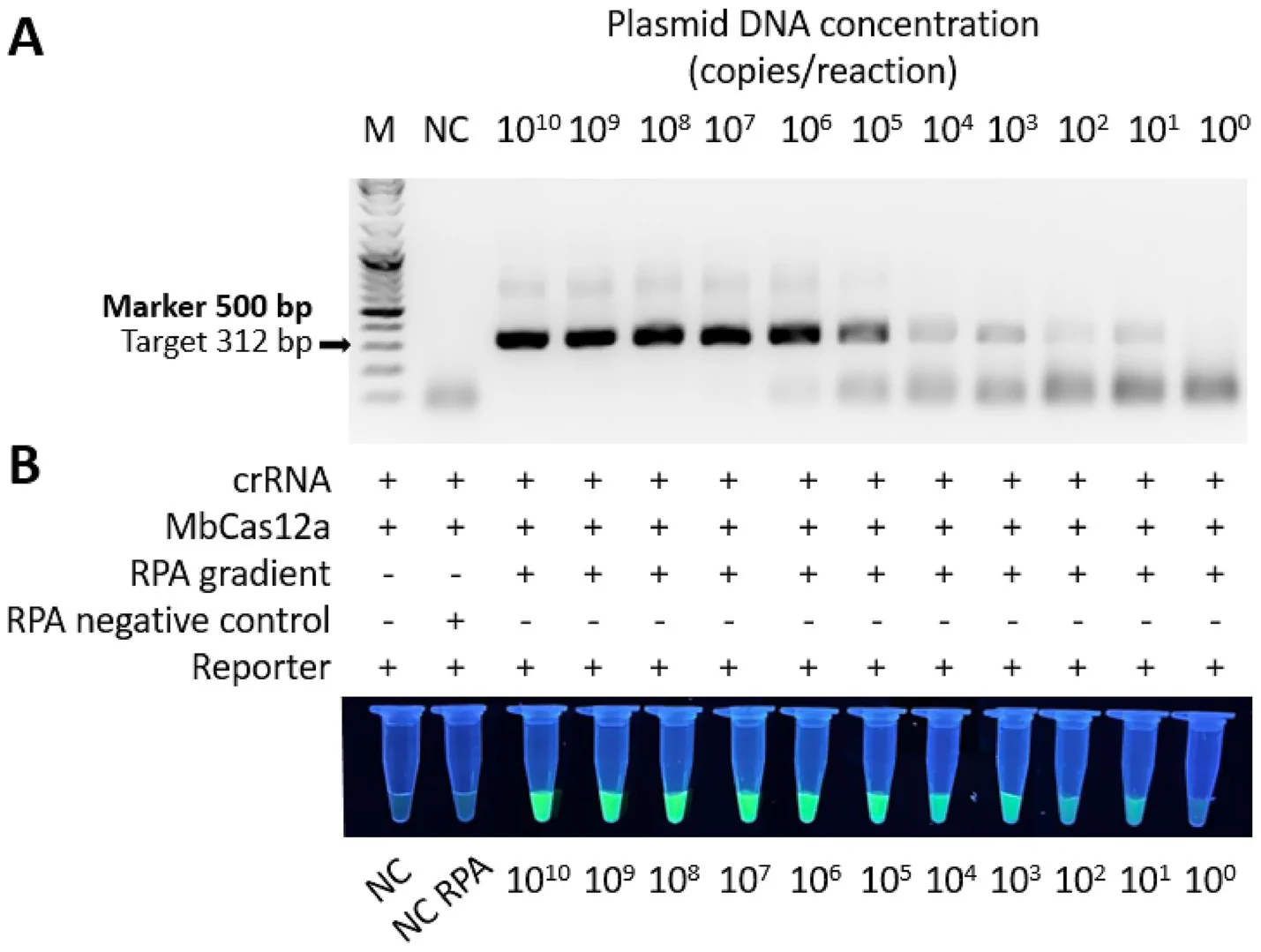
Limit of detection of the RPA–CRISPR assay targeting the *sodA* gene **(A)** Agarose gel electrophoresis of RPA products generated from serial dilutions of plasmid DNA containing the *sodA* gene fragment (target copy number per reaction: 1.6 × 10*n*) **(B)** Corresponding CRISPR-Cas12a fluorescence readout for each RPA product.

**Figure 4:**
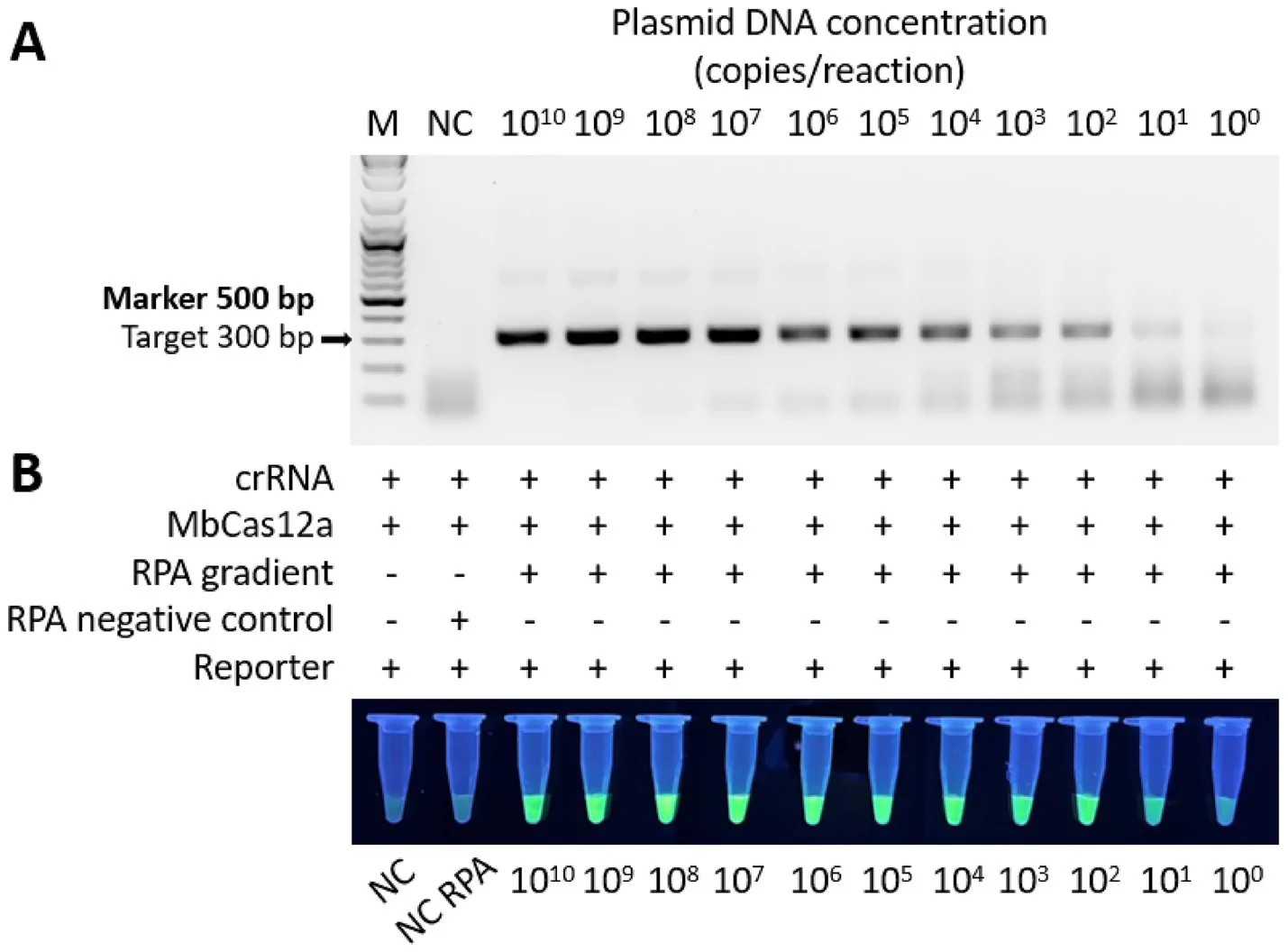
Limit of detection of the RPA–CRISPR assay targeting the *groL* gene. **(A)** Agarose gel electrophoresis of RPA products generated from serial dilutions of plasmid DNA containing the *groL* gene fragment (target copy number per reaction: 2.5 × 10*n*). **(B)** CRISPR-Cas12a fluorescence detection corresponding to the RPA products.

The analytical sensitivity of the assay was determined by establishing the limit of detection (LoD) for each genetic target. For LoD analysis, plasmid vectors containing defined fragments of the *sodA* or *groL* genes were constructed and used as DNA templates. The use of plasmid constructs enabled precise control of target copy number and ensured reproducible input concentrations across serial dilutions. Plasmid DNA templates were serially diluted to defined copy numbers and subjected to RPA amplification followed by MbCas12a-based CRISPR detection (Figure 3; Figure 4).

RPA amplification of the *sodA* target enabled reliable detection at 1.6 × 10^1^ copies per reaction, whereas amplification of *groL* was consistently detected at 2.5 × 10^1^copies per reaction. Although a weak fluorescence signal was observed at 2.5 × 10^0^copies per reaction for the *groL* target, this signal was close to the background level and was therefore not considered sufficient for defining the analytical limit of detection. Subsequent CRISPR-Cas12a readout produced fluorescence signals that corresponded to the same detection thresholds for each target, confirming concordance between amplification and CRISPR-based detection. No fluorescence above background levels was observed in no-template control reactions.

**Figure 5:**
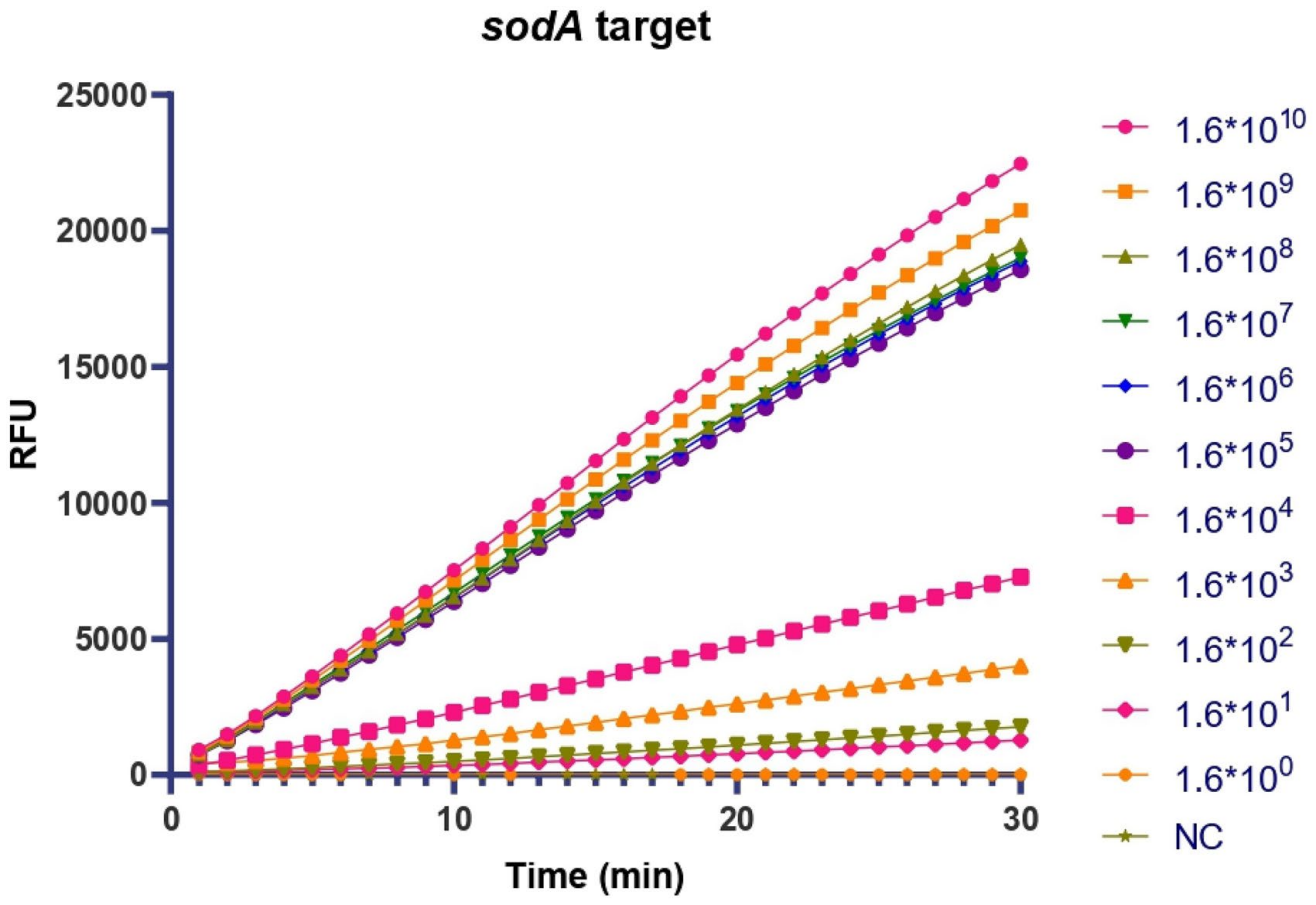
Real-time fluorescence kinetics of MbCas12a-based CRISPR detection for *sodA* target. Time-course fluorescence signals obtained during CRISPR detection using RPA-amplified products generated from serial dilutions of plasmid DNA containing the *sodA* gene fragment. RFU values were recorded over a 30-min period. Curves represent defined target copy numbers per reaction, while NC denotes no-template controls.

**Figure 6:**
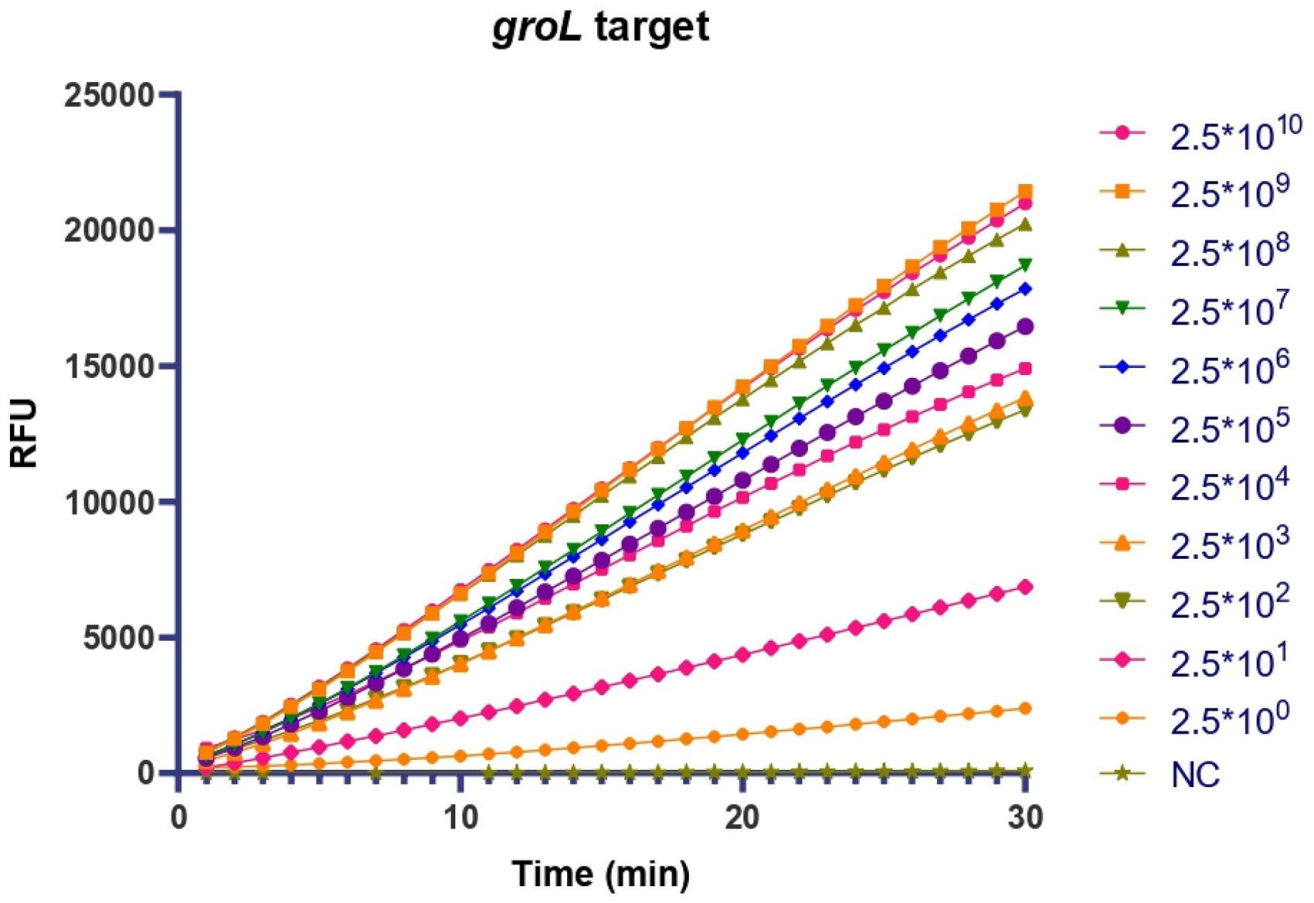
Real-time fluorescence kinetics of MbCas12a-based CRISPR detection for *groL* target. Time-course fluorescence signals obtained during CRISPR detection using RPA-amplified products generated from serial dilutions of plasmid DNA containing the *groL* gene fragment. RFU values were recorded over a 30-min period. Curves represent defined target copy numbers per reaction, while NC denotes no-template controls.

The real-time fluorescence kinetics of MbCas12a-mediated detection were further analyzed to characterize the dynamic response of the assay across a range of target concentrations for both genetic markers. RPA products generated from serial dilutions of plasmid DNA containing *sodA* or *groL* fragments were used as templates for CRISPR detection, and fluorescence signals were monitored over time to assess signal accumulation and concentration-dependent kinetics (Figure 5; Figure 6).

For both targets, fluorescence intensity increased in a time- and dose-dependent manner, with higher template concentrations producing faster signal onset and greater endpoint RFU values. The *sodA* detection demonstrated clear kinetic separation between positive reactions and negative controls, with robust signal generation across a broad range of input concentrations. Similarly, *groL* targeting assay exhibited detectable fluorescence above background at concentrations consistent with the established LoD, while lower copy numbers showed delayed or minimal signal development. No significant fluorescence increase was observed in no-template control reactions throughout the monitoring period, confirming assay specificity and stability of the CRISPR readout.

### 3.4 Detection performance on GBS isolates

**Figure 7:**
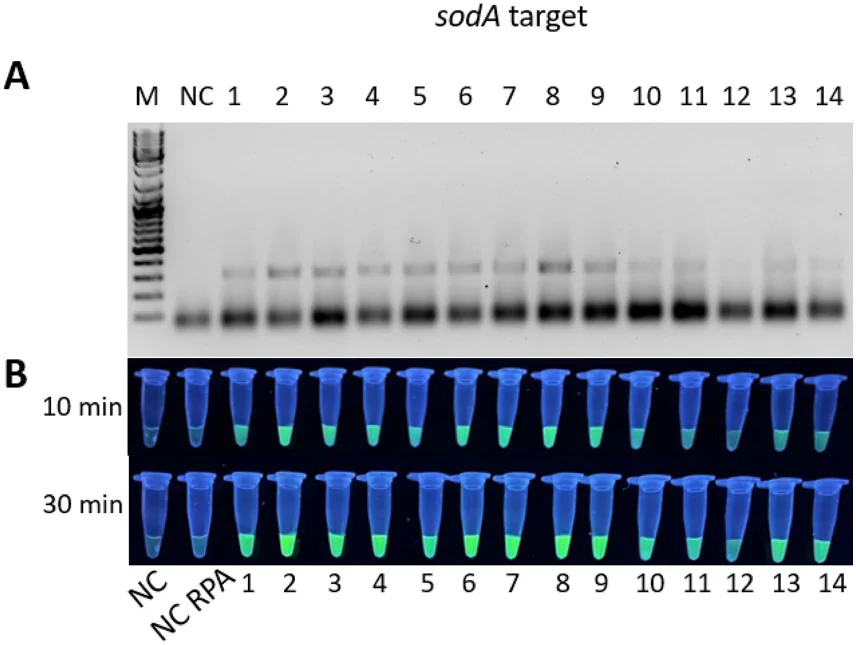
RPA-CRISPR/Cas12a assay performance on 14 GBS isolates. **(A)** Agarose gel electrophoresis of RPA amplification products with *sodA*-specific primers for 14 isolates. **(B)** Fluorescence readout of *sodA*-targeted RPA-CRISPR reactions imaged at 10 min and 30 min.

**Figure 8:**
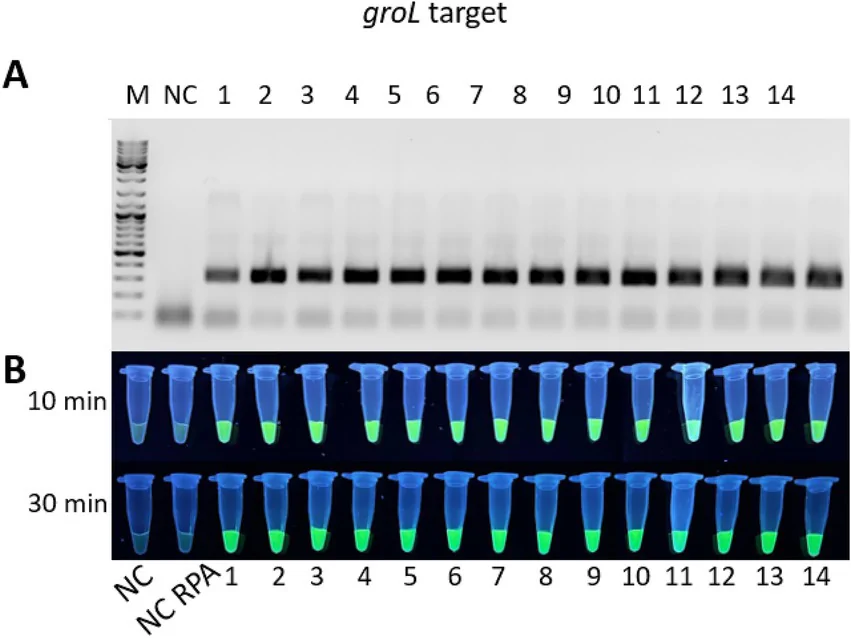
RPA-CRISPR/Cas12a assay performance on 14 GBS isolates. **(A)** Agarose gel electrophoresis of RPA amplification products with *groL*-specific primers for 14 isolates. **(B)** Fluorescence readout of *groL*-targeted RPA-CRISPR reactions imaged at 10 min and 30 min.

The RPA–CRISPR assay was evaluated under conditions compatible with rapid and low-equipment workflows using 14 GBS isolates. Bacterial material was processed using a simple heat-lysis method, generating crude lysates that could be directly used for amplification without further purification. This simplified approach reduces the time, cost, and technical requirements typically associated with nucleic acid extraction, enabling the assay to be applied in settings with limited laboratory resources. Detection was performed using two genetic targets, *sodA* and *groL* genes, allowing assessment of assay performance and robustness across different markers. The workflow and results highlight how minimally processed bacterial isolates can be effectively analyzed, while also revealing potential target-dependent differences in detection efficiency (Figure 7; Figure 8).

All 14 isolates produced detectable fluorescent signals when the *sodA* target was applied. Amplification with the *groL* target also yielded detectable fluorescence in all samples (100%), with signal intensity clearly exceeding background levels under the tested conditions. Negative controls included in each experimental run showed no detectable fluorescence, confirming the specificity of the assay and the absence of non-specific amplification. These findings demonstrate that the RPA-CRISPR assay enables reliable detection of minimally processed bacterial samples and performs consistently across all tested isolates.

## 4 Discussion

GBS continues to represent a major public health challenge. Invasive infections contribute substantially to neonatal morbidity and mortality and are increasingly affecting non-pregnant adults, particularly those with underlying comorbidities such as diabetes or advanced age. In hospital settings, GBS can cause pneumonia and soft tissue complications (Meroni et al., 2022; Coggins and Puopolo, 2024). Although intrapartum antibiotic prophylaxis has substantially reduced the incidence of early-onset neonatal invasive disease caused by GBS, late-onset disease and hospital-associated clusters in neonatal intensive care units persist, underscoring the ongoing need for rapid, sensitive diagnostic tools to enable timely intervention and effective infection control measures (Lee et al., 2024; Delettre et al., 2024). The isothermal RPA-CRISPR assay described herein, employing MbCas12a derived from *Moraxella bovoculi* for collateral cleavage-based fluorescence readout, addresses these requirements by providing a platform that operates effectively at constant moderate temperatures and integrates seamlessly with minimal sample processing.

The present study introduces several advances over previously reported CRISPR-based assays for GBS. To date, all published CRISPR assays for GBS detection have relied on the *cfb* gene as the molecular target (Yu et al., 2023; Zheng et al., 2024; Li et al., 2025; Zhao et al., 2025), whereas the present study explores two alternative housekeeping genes, *sodA* and *groL*, as CRISPR targets for the first time. Although the *cfb* locus has been widely adopted because of its association with the CAMP factor, recent studies have demonstrated important limitations of relying exclusively on this marker. In particular, CAMP-negative GBS isolates carrying complete or partial chromosomal deletions of the *cfb* gene have been reported, resulting in false-negative results in both CAMP-based identification and single-target *cfb* molecular assays. Importantly, a recently published study concluded that multitarget molecular assays substantially reduce this risk and recommended the use of multiple chromosomal targets for reliable GBS detection (Lai et al., 2025; Zhou et al., 2023). These findings provide additional justification for evaluating alternative conserved loci for CRISPR-based diagnostics.

In contrast to *cfb*, *sodA* and *groL* are conserved housekeeping genes that have long been used for phylogenetic reconstruction and species identification within the genus Streptococcus. Comparative analyses have shown that both loci provide greater taxonomic resolution than the 16S rRNA gene, with *groL* representing one of the most informative markers for discrimination of closely related streptococcal species (Glazunova et al., 2009). Building upon these characteristics, our sequence alignment analysis demonstrated complete conservation of the selected target regions among representative GBS serotypes together with multiple mismatches relative to non-target bacteria, supporting the observed analytical specificity of the assay.

Although our study did not include a direct head-to-head comparison with published *cfb*-based CRISPR assays under identical experimental conditions, the present work expands the repertoire of molecular targets available for CRISPR-based GBS diagnostics. Furthermore, by combining the recently characterized MbCas12a nuclease with a dual-gene detection strategy, our assay reduces dependence on a single genomic locus and follows the growing recommendation that molecular diagnosis of GBS should rely on multiple conserved chromosomal targets rather than a single marker (Lai et al., 2025). The assay achieved analytical limits of detection ranging from 1.6 × 10^1^ to2.5 × 10^1^ target copies per reaction and demonstrated successful detection of all tested isolates, indicating high inclusivity within the evaluated strain collection. Together, these findings support the suitability of both *sodA* and *groL* as reliable molecular targets for CRISPR-based GBS detection and establish a foundation for the further development and clinical validation of CRISPR-based diagnostic assays targeting these loci.

## 5 Conclusion

In conclusion, the RPA-MbCas12a platform developed in this study broadens the molecular toolbox available for CRISPR-based detection of GBS by combining the recently characterized MbCas12a nuclease with isothermal RPA amplification and the previously underutilized *sodA* and *groL* genes. The analytical evaluation demonstrated detection limits ranging from 1.6 × 10^1^ to 2.5 × 10^1^target copies per reaction, successful detection of all tested isolates, and compatibility with crude lysates, supporting the feasibility of a simplified and rapid workflow. Together, these findings demonstrate the potential of this dual-target MbCas12a strategy for CRISPR-based GBS detection and provide a foundation for further analytical optimization and potential point-of-care implementation, in alignment with the demands of modern infection prevention in human healthcare.

## Data Availability

The original contributions presented in the study are included in the article/Supplementary material, further inquiries can be directed to the corresponding author/s.
