# Supplementary material for: Rapid detection of Streptococcus agalactiae using an RPA-MbCas12a CRISPR assay targeting novel sodA and groL markers
Source: Front Microbiol. 2026 Aug 24;17:1918484. doi: 10.3389/fmicb.2026.1918484 (PMC13547278; doi:10.3389/fmicb.2026.1918484)
Supplement: Supplementary file 1 [file Data_sheet_1.docx]

Supplementary Material

# 1 Supplementary Data

Supplementary Material should be uploaded separately on submission. Please include any supplementary data, figures and/or tables.

Supplementary material is not typeset so please ensure that all information is clearly presented, the appropriate caption is included in the file and not in the manuscript, and that the style conforms to the rest of the article.

# 2 Supplementary Figures and Tables

For more information on Supplementary Material and for details on the different file types accepted, please see [here](https://www.frontiersin.org/guidelines/author-guidelines#supplementary-material).

## 2.1 Supplementary Figures

**Supplementary Figure 1.** Multiple sequence alignment of the PAM-groL/sodA target regions and RPA primers among representative strains of the five most prevalent GBS serotypes (Ia, Ib, II, III, V).

**Supplementary Figure 2.** Multiple sequence alignment of the PAM-groL target region and RPA primers across selected bacterial species. Asterisks (*) at the top indicate conserved positions across all aligned sequences.

**Supplementary Figure 3.** Multiple sequence alignment of the PAM-*sodA* target region and RPA primers across selected bacterial species. Asterisks (*) at the top indicate conserved positions across all aligned sequences.
